# Minimizing the hidden dangers of cholecystectomy in vascular Ehlers‐Danlos syndrome through a multidisciplinary approach

**DOI:** 10.1002/ccr3.4090

**Published:** 2021-06-10

**Authors:** Jasmine Crane, Stephen Lam, Jian Shen Kiam, Bhaskar Kumar

**Affiliations:** ^1^ Norfolk and Norwich University Hospitals NHS Foundation Trust Norwich UK; ^2^ University of East Anglia Medical School Norwich UK

**Keywords:** cholecystectomy, Ehlers‐Danlos, multidisciplinary approach, surgery

## Abstract

Ehlers‐Danlos syndrome, specifically EDS4, can be a dangerous condition. Clinicians should be aware of this when referring such patients for any interventional procedure. An MDT approach should be adopted to help plan perioperative treatment and care.

## Graphical abstract

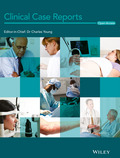

## 1 INTRODUCTION

This case report describes the perioperative planning of a woman with Ehlers‐Danlos syndrome (EDS) type IV subtype, which increases the risk of surgical bleeding complications, requiring close medical and surgical collaboration. Paramount to success was the meticulous planning of the operation with respect to minimizing the risk of anticipated complications that stem from increased tissue friability.

Ehlers‐Danlos syndrome defines a group of genetic connective tissue disorders characterized by fragile skin, easy bruising, spontaneous rupture of arteries and joint dislocation.^1^ EDS has multiple surgical risk factors described in the literature including a high risk of visceral perforation and aneurysm rupture.^2^, ^3^ The overall incidence of Ehlers‐Danlos is approximately 1 in 5000 people.^2^ EDS subtype IV (EDS4) accounts for approximately 5% of all cases.^4^

Ehlers‐Danlos syndrome subtype IV refers to an autosomal dominant vascular subtype caused by mutations of the COL3A1 gene^2^ causing a deficit of type III collagen. Type III collagen is a constituent of arterial walls and the digestive tract predisposing EDS4 patients to vascular and digestive tract ruptures.

We report a case of a patient with EDS4 who required an elective cholecystectomy for gallstone disease. EDS4 patients with gallstone disease are at an increased risk of requiring emergency surgery; therefore, elective cholecystectomy is indicated.^2^ However, minimal published information is available to the surgeon to help plan such cases.^5^, ^6^

Collaboration between upper gastrointestinal and vascular surgeons, cardiologists, and anesthetists was important in the planning of the surgery to reduce complication risk. The case identifies important considerations in planning elective operations in patients with EDS4 which may help guide clinicians who deal with similar cases in the future.

## 2 CASE HISTORY

A 47‐year‐old female engineer with known EDS4 was referred to an upper gastrointestinal surgery clinic at a tertiary academic teaching hospital having had severe episodes of biliary colic.

The patient was well known to the vascular and cardiology departments having both a stable 7 mm renal artery and a 10 mm splenic artery aneurysm and a normal aorta on annual surveillance. In addition, she had had a prior non‐ST segment elevation myocardial infarction (NSTEMI) with a dissected right coronary artery requiring four drug eluted stents and long‐term antiplatelet treatment.

## 3 INVESTIGATIONS

Blood count reported a white cell count of 10.0 × 109/L, C‐reactive protein 1 mg/dL, hemoglobin 132 g/L, platelet count 304 × 109/L, amylase 30 U/L, bilirubin 7 U/L, alkaline phosphatase (ALP) 53 U/L, alanine aminotransferase (AST) 28 U/L, with a normal kidney function. An abdominal ultrasound reported multiple gallstones with sludge in a thin‐walled gallbladder without biliary tree dilatation.

Our surgical team conducted an extensive preoperative assessment which included discussion with a regional genetic service and surgical colleagues from other tertiary centers. The first decision was whether to commit to surgical intervention or not. The decision‐making process required clear communication with the patient of the risks and benefits of intervention vs a more conservative watch and wait approach. Both patient and surgeon felt that it would be safer to intervene in a controlled elective setting rather than risk intervention should the patient develop gallstone‐related complications such as acute cholecystitis or gallstone pancreatitis. The benefits of intervention in a controlled elective manner seemed to be the most favorable approach. With regard to the timing of surgery, it was felt that it would be prudent to offer surgery at the earliest opportunity rather than delay and risk an emergency presentation.

Regarding the surgical technique and associated risks, a decision needed to be made about a laparoscopic vs open surgical approach. Due to the potential for postoperative wound herniation, both short and long term, it was felt that a standard laparoscopic approach was preferable to open surgery. Following discussion with various members from the vascular surgery team, the risk of tissue friability was highlighted particularly with respect to using automatic closure or clipping devices. It was felt that these devices may exert shear forces which could divide rather than secure tissue, for example, cystic duct. A decision was made to use Hem‐o‐lock^®^ rather than mechanical clippers since the former could be placed in a more controlled manner with less force. Alternative methods to hand, if needed, such as ligatures were also made available.

Review of computed tomography (CT) images at a vascular multidisciplinary team (MDT) showed no visible aneurysms close to the proposed operation site reducing the risk of aneurysm rupture during the operation. Arrangements were made for a vascular surgeon to be on standby during the operation in the event of any vascular rupture (eg, splenic artery aneurysm rupture). The patient's medications were discussed with a cardiology team, and Ticagrelor was stopped. Vascular cover was arranged. A HDU bed was booked in advance to observe for any acute postoperative complication. The predicted operative mortality was 4.8%, ASA grade was 3, and body mass index (BMI) was 24.5. The risks of general anesthesia (eg, airway disruption from endotracheal intubation) were also considered and arrangements made to ensure a consultant‐led pre‐assessment visit as well as senior anesthetic presence during surgery.

The patient was subsequently fully informed of any possible complications, particularly the increased risks of bleeding and bile leak from a failure to secure the cystic duct and a small but not insignificant risk of mortality.

## 4 TREATMENT

General anesthesia did not pose any complications. A standard laparoscopic port placement was performed starting with a modified Hasson port insertion with a single 11 mm infraumbilical port an 11 mm epigastric and two 5 mm lateral ports. The gallbladder was found to be contracted and inflamed with dense adhesions. Dissection of Calot's triangle was difficult due inflammation and extensive friable bleeding tissues. Strasberg's critical view of safety was achieved,^7^ and the cystic artery and duct were each secured with two Hem‐o‐lock^®^ clips. Hemostasis was secured with diathermy, and a small amount of FIBRILLAR^TM^ applied to the gallbladder fossa in the liver. A 21Fr Wallace drain was inserted at the gallbladder fossa to assess for blood loss or early bile leak. The 11 mm ports were closed with a No.1 J Polyglactin 910 (VICRYL^®^) suture. Tissue strength appeared to be satisfactory. The patient was monitored in HDU overnight and stepped down to the ward. The patient remained stable and clinically well and was therefore discharged on day two postoperatively. The drain remained in situ for a further week postdischarge as a precaution.

## 5 OUTCOME AND FOLLOW‐UP

The patient required no further follow‐up for the laparoscopic cholecystectomy but has ongoing annual aneurysm surveillance, demonstrating approximately 2 mm growth over a 2‐year period. The right renal artery aneurysm appears unchanged at 9 mm, the splenic artery aneurysm unchanged at 15 mm, normal caliber of the femoral and popliteal arteries. There is an unchanged left internal carotid artery and bulb dilatation to 10 mm which again is unchanged.

## 6 DISCUSSION

Ehlers‐Danlos Syndrome (EDS) describes a group of inherited connective tissue disorders that affect collagen synthesis with different subtypes. EDS affects approximately 1 in 5000 people worldwide.^4^ The cardinal features include joint hypermobility, skin laxity, hyperextensibility, and tissue fragility. These features amount to significant risks of major postoperative complications including mortality.^1^

Surgical intervention in patients with Ehlers‐Danlos syndrome is challenging. A high frequency of surgical complications has been described in the literature, especially from the vascular subtype (EDS4)^2^, ^4^ Patients with EDS4 are prone to spontaneous rupture of visceral organs, aneurysms, and dissections with an increased mortality rate.^2^, ^3^, ^4^ In addition, EDS patients pose a further anesthetic risk of cervical atlantoaxial subluxation as a consequence of the laxity of the cervical ligaments, as well as temporomandibular dislocation during endotracheal intubation.^8^

In our experience with this patient, successful surgical outcome was achieved through meticulous preoperative preparation aimed at minimizing the risks involved. In this report, we detail the steps we have taken which may be useful for all clinicians dealing with these patients in the future. This is also the first report in the literature detailing the steps of successful outcome of laparoscopic cholecystectomy in a patient with EDS4.

## CONFLICT OF INTEREST

All authors have disclosed no conflict of interest.

## AUTHOR CONTRIBUTIONS

JC: drafted the manuscript and contributed to the study, critically revised the manuscript, and approved the final version for publication. SL: contributed to the concept and design of the study, acquisition of figures, critical revision of the manuscript and approved the final version. JSK: contributed to the critical revision of the manuscript and approved the final version. BK: contributed to the concept and design of the study, critical revision of the manuscript, acquisition of photos and approved the final version.

## ETHICAL APPROVAL

Ethical approval was not required for this study.

## Data Availability

The data supporting the findings of this study are available within the article.
